# Structural Identification and Antioxidant Activity of Loach Protein Enzymatic Hydrolysates

**DOI:** 10.3390/molecules28114391

**Published:** 2023-05-28

**Authors:** Jinrong Mao, Shunqin Li, Liyuan Yun, Min Zhang

**Affiliations:** 1 China-Russia Agricultural Processing Joint Laboratory, Tianjin Agricultural University, Tianjin 300384, China; m18087825705@163.com; 2 State Key Laboratory of Food Nutrition and Safety, Tianjin University of Science and Technology, Tianjin 300457, China; sqli18306891445@163.com

**Keywords:** loach, bioactive peptide, antioxidation, structural identification

## Abstract

Loach, rich in nutrients, such as proteins, amino acids, and mineral elements, is being gradually favored by consumers. Therefore, in this study, the antioxidant activity and structural characteristics of loach peptides were comprehensively analyzed. The loach protein (LAP) with a molecular weight between 150 and 3000 Da was graded by ultrafiltration and nanofiltration processes, which exhibited excellent scavenging activity against DPPH radical (IC50 2.91 ± 0.02 mg/mL), hydroxyl radical (IC50 9.95 ± 0.03 mg/mL), and superoxide anion radical (IC50 13.67 ± 0.33 mg/mL). Additionally, LAP was purified by gel filtration chromatography, and two principal components (named as LAP-I and LAP-II) were isolated. A total of 582 and 672 peptides were identified in LAP-I and LAP-II, respectively, through structural analysis. The XRD results revealed that LAP-I and LAP-II had an irregular amorphous structure. The 2D-NMR spectroscopy results suggested that LAP-I had a compact stretch conformation in the D_2_O solution, while LAP-II had a folded conformation. Overall, the study results suggested that loach peptide could be a potential antioxidant agent and might provide valuable information for chain conformation and antioxidant mechanism research further.

## 1. Introduction

Loach is a prevalent freshwater fish and is widely distributed in China, Korea, Japan, and other East Asian countries. It serves as a kind of tender, tasty, and nutritious food with a long history of medicinal uses in China [1]. It is often used for treating various diseases, such as acute infectious hepatitis jaundice, osteomyelitis, and cancers, and for restoring health [2]. Loach is also called “ginseng in the water” due to its richness in nutrients and medicinal value [3]. Recent studies showed that lectin, peptide, and polysaccharides purified from loach or its mucus pose significant biological activities [1,4,5]. Loach peptides attracted considerable interest due to their numerous biological activities. Dong, X.Z et al. reported that the peptides isolated from loach had good antibacterial activity, which could inhibit various bacteria, such as *Bacillus subtilis*, *Staphylococcus aureus*, and *Escherichia coli* [6]. In the study of ACE inhibitory activity, the loach peptide significantly decreased the systolic blood pressure with an IC50 of 18.2 ± 0.9 μg/mL [1]. Liu, H et al. found that loach skin collagen peptides and sodium selenite could improve bone microarchitecture and maintain the blood calcium level and phosphate homeostasis in mice [7]. You, L et al. found that the loach peptides had excellent DPPH radical scavenging activity, with an IC50 17.0 ± 0.54 mg/mL and hydroxyl radicals scavenging activity with an IC50 2.64 ± 0.29 mg/mL, which could improve the endurance capacity and promote fatigue recovery in mice [8].

Natural antioxidant peptides are a type of short peptide which are safe, rich in amino acids, and easily absorbed by the human body. As such, the development and application of natural antioxidant peptides received increasing attention. Particularly, the antioxidant properties of fish hydrolysis products became a topic of interest in health foods, pharmaceuticals, and food processing and preservation industries [9]. Generally, in vitro chemical methods or in vivo experimental methods are used to determine the antioxidant activity of natural peptides. Wang et al. [10] found that the cottonseed peptide with a molecular weight < 3 kDa had strong antioxidant activity, the DPPH free radicals scavenging activity with an EC50 0.49 ± 0.02 mg/mL, ABTS free radicals scavenging activity with an EC50 2.05 ± 0.02 mg/mL, and hydroxyl radical scavenging activity with an EC50 2.21 ± 0.12 (EC50 is the half maximum effect concentration; when it is used to describe the rate of inhibition, the smaller the value, the more effective). Additionally, the walnut peptides showed excellent protective effects against H_2_O_2_-induced oxidative stress in the PC-12 cells in a dose-dependent manner. These results support the hypothesis that the antioxidant activity of natural peptides is closely related to their structural characteristics. Ke H.L. et al. identified a total of 25 peptides from oyster shell chelated peptides by LC-MS/MS and found that Glu, Asp, and Gly residues had the high calcium-binding ability [11].

In recent years, functional foods became popular worldwide due to their great potential as functional ingredients in food and health applications [12,13]. Peptides derived from animal sources are often used in several products, such as food, cosmetics, pharmaceutical/biomedical products, and nutritional products [14]. Notably, as a health food, the antioxidant peptide has the potential to delay aging, and reduce the risk of inflammation and cardiovascular diseases, consequently improving human health [15]. Moreover, the application of antioxidant peptides in the food industry is beneficial to retard food discoloration and deterioration caused by oxidative processes [16]. It can be used as an additive in food. The characterization, such as the amino acid sequence of a peptide, is the basis for the comprehensive understanding of active peptides [17,18]. Unfortunately, the sequence of loach antioxidant peptide, the crystal morphology of the main components of loach peptide, and its conformation in water are still unclear.

Therefore, the present study sought to investigate the antioxidant activities, sequences, and structural characteristics of loach peptides. The antioxidant activities of loach peptides were assessed by the total antioxidant activity, DPPH radical scavenging activity, hydroxyl radical scavenging ability, and superoxide anion scavenging ability. Subsequently, the loach peptides were purified by gel filtration chromatography. The structural characteristic of purified loach peptide, including molecular weight, amino acid sequences, and morphological features, were evaluated by HPLC, LC-MS/MS, Fourier transform infrared (FT-IR) spectroscopy, X-ray diffraction, and 2D Nuclear Magnetic Resonance. Based on the antioxidant activity and structural morphology of LAP, it can be applied to functional foods, nutritional regulators, and food preservation in future research and application.

## 2. Results and Discussion

### 2.1. Separation of LAP Using Gel Filtration Chromatography

**Figure 1:**
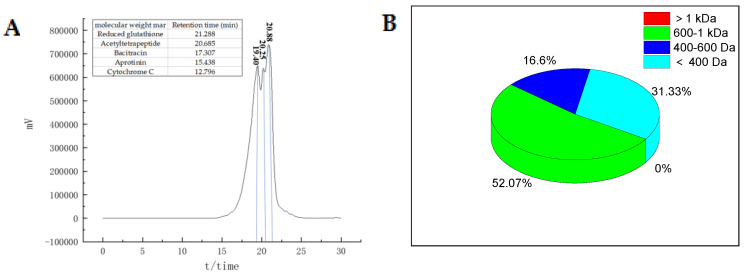
Molecular weight map of LAP. (**A**) Molecular mass distribution of LAP; (**B**) Percentage of LAP molecular mass distribution.

Accumulating studies reported that the molecular weight (MW) of peptides is closely related to their antioxidant activity, and lower MW peptides have higher antioxidant capacity [19,20]. For instance, Sarmadi et al. [18] found that peptides with MW between 500 and 1000 Da exhibited stronger antioxidant activity than those with MW > 1500 Da or <500 Da. Similarly, Anwar et al. [21] found that hydrolysates from Chinese sturgeon protein containing low molecular weight (<1000 Da) components displayed the highest antioxidant activity. The molecular weight map of LAP is presented in Figure 1A, which shows that peptides with MW < 1000 Da account for 99.72% of the total peptides (Figure 1B). This finding was consistent with the reported characteristics of bioactive peptides, suggesting that the antioxidant activity of LAP might be attributed to the low MW peptides.

### 2.2. Antioxidant Activity of LAP

**Figure 2:**
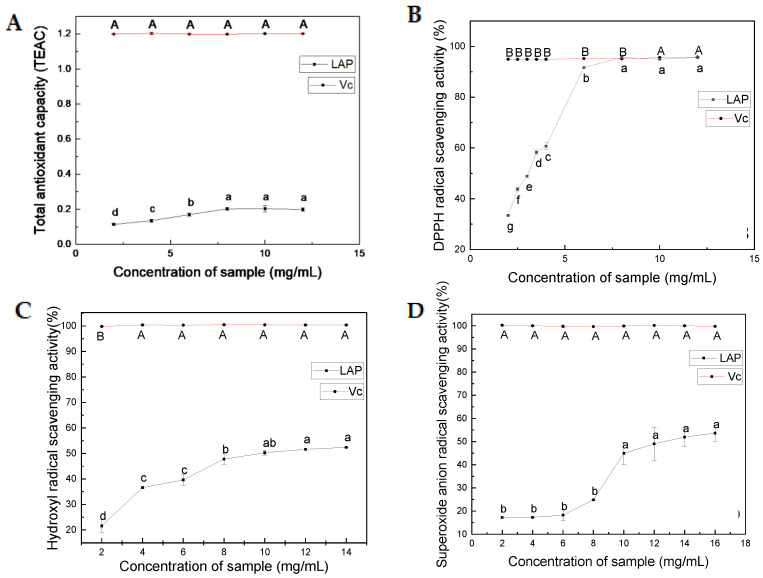
Antioxidant activity of LAP. (**A**) The total antioxidant activity; (**B**) DPPH radical scavenging; (**C**) hydroxyl radical scavenging; (**D**) superoxide anion radical scavenging. **Note:** Values are expressed as mean ± SD, n = 3, different letter marks indicate significant differences, *p* < 0.05.

The antioxidant activity of LAP was determined by assessing its total antioxidant activity, DPPH radical scavenging rate, hydroxyl radical scavenging rate, and superoxide anion scavenging rate at different concentrations (2–12 mg/mL), and the results are presented in Figure 2A–D. The IC50 value was used to assess the antioxidant activity of LAP, and lower IC50 values indicate higher free radical scavenging effects [22].

#### 2.2.1. Total Antioxidant Activity

Trolox was used as a standard product to express the total antioxidant activity of LAP in terms of its antioxidant capacity compared to Trolox at the same concentration, while Vc (Vitamin C) served as the positive control. The total antioxidant activity gradually increased with increased LAP concentration and reached a maximum value at 10 mg/mL, which was equivalent to a Trolox total antioxidant capacity of 0.20354 ± 0.0188 mM (Figure 2A).

#### 2.2.2. DPPH Radical Scavenging Activity

DPPH radical is a stable free radical and is used as a measure for radical scavenging activity of natural antioxidants [23]. Vc was used as the positive control. The results (Figure 2B) showed that the DPPH radical scavenging was stable at 95% with increased Vc concentration. However, the DPPH radical scavenging rate showed an upward and then flattening trend with increased LAP concentration. When the concentration of LAP exceeded 8 mg/mL (with an IC50 value of 2.91 ± 0.02 mg/mL), the DPPH radical scavenging activity similar to Vc showed that LAP had strong DPPH radical scavenging activities.

#### 2.2.3. Hydroxyl Radical Scavenging Activity

The hydroxyl radical is an extremely reactive free radical in biological tissue proteins, which can easily react with lipids, amino acids, proteins, DNA, and other biological molecules in living organisms, inducing physiological disorders [24,25]. Hydroxyl radical inhibition activity (Figure 2C) increased in a dose-dependent manner with increased LAP concentration until the concentration exceeded 10 mg/mL, after which it gradually decreased with an IC50 value of 9.95 ± 0.03 mg/mL.

#### 2.2.4. Superoxide Anion Radical Scavenging Activity

Superoxide anion radical is a signaling molecule, which can promote oxidation reaction, and acts as a key factor in regulating apoptosis and aging [26,27]. The superoxide anion radical inhibition activity (Figure 2D) increased in a dose-dependent manner with increased LAP concentration, with an IC50 value of 13.67 ± 0.33 mg/mL.

These results suggest that LAP possesses strong antioxidant activity. In this study, the DPPH radical scavenging activity of LAP was higher than the loach peptide prepared by You et al., with an IC50 value of 17.0 ± 0.54 mg/mL [8]; the skipjack tuna peptide (STG-AH-I) at 10 mg/mL, the DPPH radical scavenging was 56.49 ± 0.41% [28], and fish industry visceral waste peptide with an IC50 value of 3.47 mg/mL [9,29]. This might have happened due to the different enzymatic hydrolysis methods.

### 2.3. Amino Acid Sequencing

**Figure 3:**
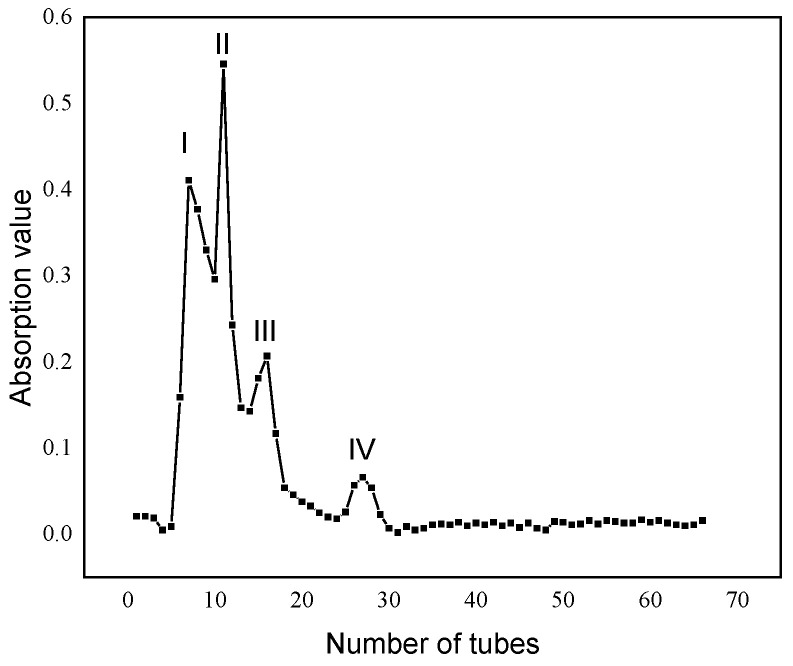
Elution profile of LAP on gel G-15. **Note:** I: LAP-I component; II: LAP-II component; III: LAP-III component; IV: LAP-IV component.

Gel chromatography is an effective method for separating samples. The LAP based on MW distribution was purified using the Sephadex G-15 medium for gel filtration. LAP was separated into four fractions: LAP-I, LAP-II, LAP-III, and LAP-IV (shown in Figure 3). The mass percentage of each component was 81.28%, 16.84%, 1.33%, and 0.55%, respectively. The principal component fractions were LAP-I and LAP-II.

**Figure 4:**
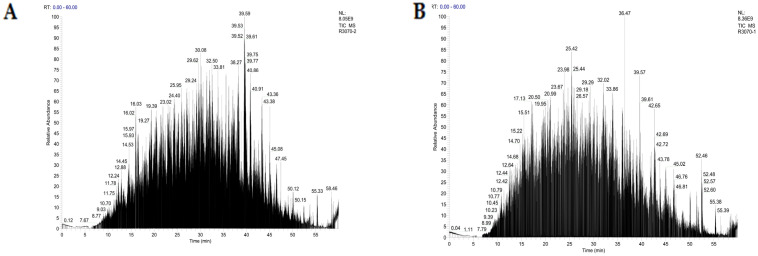
The total ion flow diagram of LAP principal components fractions. (**A**) LAP-I; (**B**) LAP-II.

Amino acid sequence analysis with LC-MS/MS was conducted for LAP-I and LAP-II. A total of 582 peptides were identified in LAP-I and 672 peptides were identified in LAP-II. Among the two components, the peptides with a molecular weight between 400 and 1000 Da accounted for 55.00% and 68.90% of the identified peptides, respectively. The primary mass spectra are shown in Figure 4A,B.

These peptide segments were prescreened based on the standard score, relative abundance, and sequence characteristics. After prescreening, 35 peptides with high confidence were obtained and are listed in Table 1.

**Figure 5:**
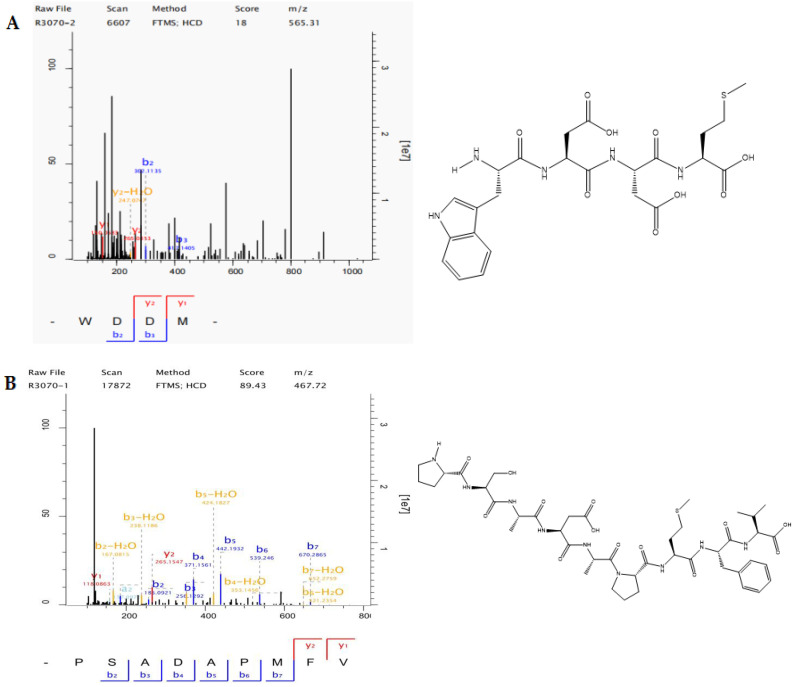
Secondary mass spectra of WDDM and PSADAPMFV peptides in the LAP principal components fractions. (**A**) WDDM; (**B**) PSADAPMFV.

Wong et al. [30] reported that in silico bioinformatics methods can identify new peptides and predict their activity, toxicity, and isoelectric point. Therefore, the probabilities for bioactivity of these peptides derived from the LAP principal components were predicted by the PeptideRanker program, using the threshold value of >0.8 for the PeptideRanker score [30,31]. The two potentially bioactive peptides screened from the LAP principal components. The prediction of toxicity and PI of these peptides was performed using PeptideRanker. The two peptides were WDDM and PSADAPMFV, which are listed in Table 2, and the secondary mass spectra are shown in Figure 5A,B.

Previous studies showed that the antioxidant ability of bioactive peptides is related to their amino acid sequences and composition [32]. Peptides containing Gly (G), Leu (L), Pro (P), Val (V), and Ala (A) showed strong antioxidant activity [33]. Furthermore, the number and location of aromatic amino acids, hydrophobic amino acids, sulfur-containing amino acids, and basic amino acids could contribute to their antioxidant activities [34]. In this study, the hydrophobic amino acid content of peptide WDDM was 50%, while the peptide PSADAPMFV was 77.78%. The N-terminal hydrophobic amino acids were consistent with the typical profile of antioxidant peptides [34]. The Ile-Ile-Ala-Pro-Pro-Glu-Arg (IIAPPER) [35], Ala-Gly-Pro-Ser-Ile-Val-His (AGPSIVH) [36], and the ValLys-Val-Gly-Asn-Glu-Phe (VKVGNEF) [37] were reported to have good antioxidant activities. The hydrophobic amino acid contents were 71.43%, 57.14%, and 42.86%, respectively, and the N-terminal amino acid was hydrophobic amino acid. Therefore, it was inferred that WDDM and PSADAPMFV could exhibit strong antioxidant activity. In future experiments, we aim to synthesize these two peptide segments and explore their antioxidant activity.

### 2.4. Morphological Analysis

#### 2.4.1. FT-IR Analysis

**Figure 6:**
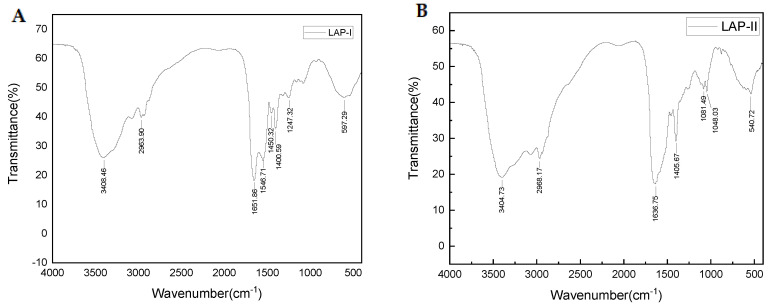
FTIR spectrogram of LAP principal components. (**A**) LAP−I; (**B**) LAP−II.

The FT-IR spectra of LAP-I and LAP-II are shown in Figure 6A,B. The results showed that the amide A band of LAP-I and LAP-II appeared at 3408.46 and 3404.73 cm^−1^, respectively, indicating a stretching vibration for free N–H [38]. The amide B band of LAP-I and LAP-II appeared at 2963.90 and 2968.17 cm^−1^, respectively, indicating an asymmetrical stretch for CH_3_ [38]. The amide I band of LAP-I and LAP-II appeared at 1657.86 and 1636.75 cm^−1^, respectively. LAP-I exhibited an α-helix characteristic absorption peak in the amide I region, while LAP-II exhibited a β-pleated sheet absorption peak in the amide I region [39]. The amide II band of LAP-I appeared at 1546.71 cm^−1^, which was mainly caused by the NH bend coupled with CN stretch [40]. LAP-I had amide III absorption peaks, indicating random coil absorption peaks at 1247.32 cm^−1^ [39]. LAP-II had fingerprint region peaks [11] at 1081.49 and 1048.03 cm^−1^, respectively, mainly caused by C-H vibration and -OH deformation in the carboxyl group [41]. The peak value of LAP was similar to Ke et al., [11] indicating that the amide A, amide I, and amide II of HSM (anchovy hydrolysate) appeared at 3041.19 cm^−1^, 1624.25 cm^−1^, and 1542.97 cm^−1^, respectively. The fingerprint region peak appeared at 1080.85 cm^−1^. Therefore, the LAP principal components with the characteristic properties of peptides [39,42,43].

#### 2.4.2. XRD Analysis

**Figure 7:**
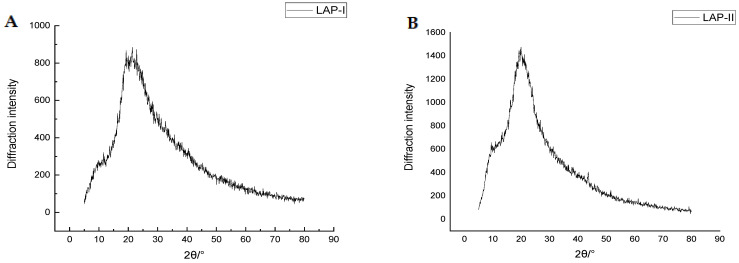
XRD spectra of LAP principal components. (**A**) LAP-I; (**B**) LAP-II.

The XRD spectra of LAP-I and LAP-II exhibited a hump shape (Figure 7A,B). No strong diffraction peaks were observed for LAP-I and LAP-II, indicating that both LAP-I and LAP-II had an irregular amorphous structure [44]. The diffraction peaks of LAP-I and LAP-II were 2θ = 20.771° and 19.984°, respectively. Usually, the smaller dÅ value indicates a more compact structure [45]. The dÅ value of LAP-I (2.1715 Å) was lower than LAP-II (2.2528 Å), indicating that LAP-I had a more compact structure than LAP-II. As the peptide molecular weight decreased, the diffraction peaks of each component remained unchanged. This was consistent with the results of Zhang et al., reporting the diffraction peaks of different molecular weight *Sporisorium reilianum* polypeptides [39]. The diffraction peaks of both LAP-I and LAP-II were consistent with the previous studies having the same diffraction angle [12,39]. Therefore, it can be inferred that this diffraction peak is the characteristic diffraction peak of peptide substances. Therefore, XRD data also indicated that LAP-I and LAP-II were in line with the characteristics of peptide substances.

#### 2.4.3. 2D-NMR Analysis

**Figure 8:**
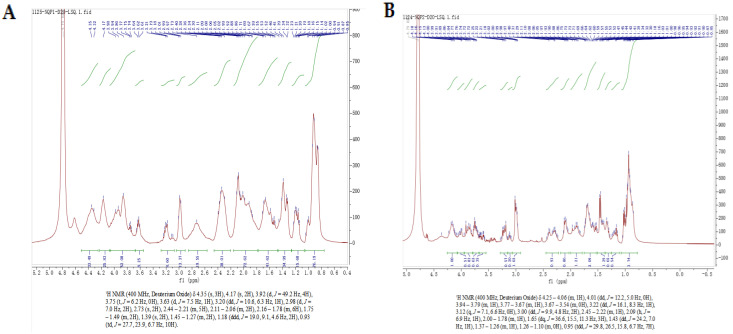
^1^H-NMR spectrum of LAP principal components. (**A**) LAP-I; (**B**) LAP-II.

**Figure 9:**
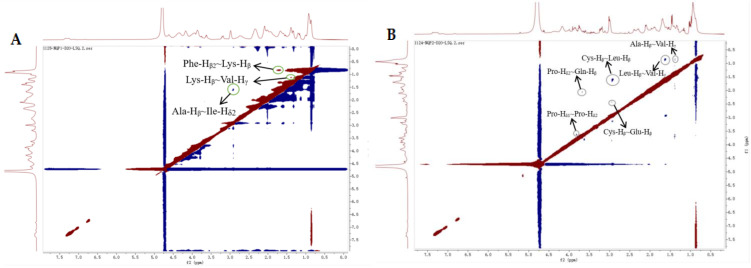
NOESY profile of LAP principal components. (**A**) LAP-I; (**B**) LAP-II.

2D-NMR spectroscopy is the most reliable technique to access information on the secondary structure and interactions of peptides in a solution [46]. 2D-NOESY spectrum could provide information on two non-bonded protons [46]. The appearance of an NOE peak is evidence that the two protons are less than 5 Å apart from each other in space [47]. The ^1^H-NMR and NOESY experiment results of LAP-I and LAP-II in D_2_O are presented in Table 3, Figure 8 and Figure 9. In D_2_O solution, the LAP-I showed three NOE signals, including Lys-H_β_ and Val-H_γ_, Phe-H_β2_ and Lys-H_β_, and Ala-H_β_ and Ile-H_δ2_. None of these three NOE signals were attached to the same C atom, indicating the compact stretch state of LAP-I in D_2_O [48,49]. LAP-II showed six NOE signals, including Ala-H_β_ and Val-H_γ_, Leu-H_β_ and Val-H_γ_, Cys-H_β_ and Leu-H_β_, Cys-H_β_ and Glu-H_β_, Pro-H_δ2_ and Gln-H_β_, and Pro-H_δ1_ and Pro-H_δ2_. The hydrogen atoms Pro-H_δ1_ and Pro-H_δ2_ produced NOE signals with each other, indicating the “folded” state in LAP-II [48,49]. Different substances exhibit different states in water due to the properties of the substances. It might be attributed to the fact that the two chains connected by the disulfide linkage of the peptide molecules exhibit different flexibilities in water [48]. Qi et al. [49] reported that GEF (Gly Glu Phe) usually exists in a stretched state in water. LAP-I was similar to GEF, showing a stretched state in water, and LAP-I was more compact. Zhang et al. [48] reported that GSSG (the oxidized glutathione) had a certain folding conformation in water. LAP-II was similar to GSSG, showing the “folded” state in water. Therefore, it can be inferred that the flexibility of LAP-I and LAP-II peptide chains is different.

## 3. Materials and Methods

### 3.1. Materials

Loach was obtained from the Tianjin Hongqi Agricultural Trade Market, Tianjin, China. Alkaline protease (134,017.1 U/g) was acquired from Beijing Solebo Technology Co., Ltd., Beijing, China. The T-AOC Assay Kit, DPPH radical scavenging activity assay kit, and Hydroxyl radical scavenging activity assay kit were acquired from Nanjing Jiancheng Biological Research Institute, Jiangsu, China. All chemicals used in this study were of analytical grade.

### 3.2. Extraction of Loach Peptides (LAP)

Loach was washed with water, the heads and viscera were separated, rinsed with distilled water, and then ground into minced meat. Then, it was dried at 45 °C, powdered, and set in a desiccator.

Enzymatic hydrolysis conditions: material to liquid ratio (1:10), alkaline protease (248 U/mL) at the condition of pH 10.0, the temperature at 60 °C (in the water bath) for 1.5 h. (The enzymolysis condition was the result of our laboratory’s previous research.) Later, it was cooled to room temperature and centrifuged at 5000 r/min for 20 min to remove the deposits and obtain the loach protein hydrolysate. Finally, the supernatant was graded by ultrafiltration and nanofiltration. The fraction of molecular weight ranging from 150 Da to 3000 Da was collected and freeze-dried.

### 3.3. Molecular Weight Determination

The molecular weight of LAP was determined by HPLC (Shimadzu Corporation, LC-20, kyoto, Japan) equipped with a TSKgel G2000 SWXL column (7.8 mm × 300 mm) and a UV detector (detection wavelength set at 220 nm) [7,50]. The mobile phase was 55% ultrapure water and 45% acetonitrile with 0.1% TFA trifluoroacetic acid. The column was calibrated with standards Cytochrome C (13,000 Da), aprotinin (6511.44 Da), bacitracin (1422.69 Da), acetyl tetrapeptide (539.53892 Da), and GSH (307.33 Da).

### 3.4. Antioxidant Activity Analysis

#### 3.4.1. Total Antioxidant Activity Assay

The total antioxidant activity of LAP was determined by the ABTS method using the antioxidant capacity assay kit (T-AOC Assay Kit) [51]. Vc was used as the positive control. LAP (2–12 mg/mL) and Vc (2–12 mg/mL) were mixed with the detection buffer, ABTS solution, Peroxide solution, and peroxidase, respectively. The absorbance was detected at 405 nm using a microplate reader. Taking Trolox as a standard product, the standard curve was plotted. The antioxidant capacity of LAP and Vc (expressed by the equivalent of Trolox) was obtained by substituting the absorbance values of the samples into the standard curve equation. The standard curve equation was: y = −0.8362x + 1.2775R^2^ = 0.998.

#### 3.4.2. Determination of DPPH Radical Scavenging Activity

The DPPH radical scavenging activity assay was performed according to the instructions supplied with the DPPH radical scavenging activity assay kit. The sample solutions (2–12 mg/mL) were mixed with the DPPH solution and anhydrous ethanol, followed by incubation in the dark for 0.5 h. Finally, it was centrifuged at 4000 r/min for 5 min to remove the deposits. The absorbance (A) was measured at 517 nm using a UV-1800 spectrophotometer (Beijing General Instrument Co. Ltd., Beijing, China). Vc was used as the positive control. The blank group and control group were provided by the DPPH radical scavenging activity assay kit. The DPPH radical scavenging activity was determined by the following Equation [42]:

$$\mathrm{DPPH}\;\mathrm{radical}\;\mathrm{scavenging}\;\mathrm{activity}\left(\%\right)=1-\frac{A_{2}-A_{1}}{A_{0}}\times 100\%$$

where A_0_ is the absorbance of the blank, A_1_ is the absorbance of the control group in the kit, A_2_ is the absorbance of the sample.

#### 3.4.3. The Hydroxyl Radical Scavenging Activity Determination

The hydroxyl radical scavenging activity assay was performed according to the instructions supplied with the hydroxyl radical scavenging activity assay kit. The sample solutions (2–12 mg/mL) were mixed with the substrate application solution and reagent application solution, followed by incubation for 1 min at 37 °C and the addition of chromogenic agent to terminate the reaction. Afterward, the mixture was incubated for 20 min at room temperature. The absorbance (A) was measured at 550 nm using a UV-1800 spectrophotometer (Beijing General Instrument Co. Ltd. UV-1800, China). Vc was used as the positive control. The blank group and control group were provided by the hydroxyl radical scavenging activity assay kit. The hydroxyl radical scavenging activity was determined by the following Equation:

$$\mathrm{Hydroxyl}\;\mathrm{radical}\;\mathrm{scavenging}\;\mathrm{activity}\left(\%\right)=\frac{A_{1}-A_{2}}{A_{1}-A_{0}}\times 100\%$$

where A_0_ is the absorbance of the blank, A_1_ is the absorbance of the control group in the kit, and A_2_ is the absorbance of the sample.

#### 3.4.4. The Superoxide Anion Scavenging Activity Determination

The superoxide anion scavenging activity was determined referring to the method of Lihua [42], with slight modifications. The sample solutions (2–12 mg/mL) were mixed with 0.05 mol/L of Tris-HCl buffer (pH 8.2), followed by incubation for 10 min in a water bath at 25 °C. Then, 0.1 mL of pyrogallol solution (preheated in a water bath at 25 °C) was added and incubated for 4 min. HCl (0.5 mol/L) was added to terminate the reaction. The absorbance (A) was measured at 320 nm using a UV-1800 spectrophotometer. Vc was used as the positive control. Distilled water was replaced with the sample solution for the blank determination, and distilled water was replaced with the pyrogallol solution for the control group determination. The superoxide anion scavenging activity was determined by the following Equation:

$$\mathrm{Superoxide}\;\mathrm{anion}\;\mathrm{scavenging}\;\mathrm{activity}\;\left(\%\right)=\frac{A_{0}-\left(A_{2}-A_{1}\right)}{A_{0}}\times 100\%$$

where A_0_ is the absorbance of the blank, A_1_ is the absorbance of the control group, and A_2_ is the absorbance of the sample.

### 3.5. Purification and Fractionation of LAP

Approximately 2 mL of LAP (30 mg/mL) was passed through a Sephadex-G15 column (Ø1.6 cm × 100 cm) and eluted with distilled water at a flow of 1.0 mL/min. The eluate was monitored by an ultraviolet spectrophotometer at 280 nm [52]. The principal components were concentrated and lyophilized.

### 3.6. LC-MS/MS Analysis

The principal components of LAP gel fractions were identified by liquid chromatography-tandem mass spectrometry (HPLC–MS/MS) using a Q Exactive mass spectrometer (Q Exactive, Thermo Fisher, Waltham, MA, USA). The LC-MS/MS method was performed following the methods of Cai et al. [53] with slight modifications. About 1 mg/mL of LAP principal components was loaded onto an RP-C18 column (Column Technology Inc., 0.15 mm × 150 mm) and balanced with 95% solvent A (0.1% formic acid aqueous solution). The sample solution was then sent to Zorbax 300SB-C18 peptide traps (Agilent Technologies, Wilmington, NC, USA) and separated by a liquid chromatography column. The elution program consisted of the solvent B (84% acetonitrile, 0.1% formic acid) concentration increasing from 4% to 50% over a period of 50 min, followed by a concentration from 50% to 100% over a period of 54–60 min. Data analysis was performed using MaxQuant 1.5.5.1 software and the NCBI_Cobitidae database. The screening criteria for reliable identification of peptides were set at *p* ≤ 0.01.

### 3.7. FT-IR Analysis

The secondary structure of LAP-I and LAP-II was determined using the Fourier transform infrared (FT-IR) spectrophotometer (VECTOR 20, Bruker, Salbuluken City, Germany). The dried LAP-I and LAP-II powders (1 mg) were mixed with dried KBr powder (150 mg), respectively, and pressed into thin slices using a tablet press. The wavenumber range was 400–4000 cm^−1^ (VECTOR 20, Germany) [39].

### 3.8. XRD Analysis

The XRD method was performed according to the method reported by Xie et al. [54] with slight modifications. LAP-I and LAP-II were milled through a 200-mesh sieve, respectively, and immediately determined by X-ray (DMAX2500, Rigaku, Tokyo, Japan). The scanning angle was set at 5–80°, using a step size of 0.02° with a scanning speed at 5°/min at 40 kV and 40 mA. The crystalline type of the LAP gel principal components was analyzed by MDI-Jade 6.0 software. The repeat spacing distance (dÅ) was calculated following the method of Zhu et al. [45].

$$dÅ=\frac{\lambda }{2sin\theta }\;(\lambda =1.54Å)$$

### 3.9. 2D NMR Analysis

Ten milligrams of dried LAP-I and LAP-II were dissolved in one milligram of deuteroxide (D_2_O), respectively. The 2D NMR was performed using an NMR instrument (Brucker, AVIII400M, Leipzig, Germany) [42,49]. The ^1^H and NOESY profiles of LAP-I and LAP-II were collected and analyzed by MestReNova 14.0 software.

### 3.10. Statistical Analysis

All experimental data were analyzed by one-way ANOVA using IBM SPSS 25.0 Software (SPSS, Inc., Chicago, IL, USA). The differences between the average values were considered significant at *p* < 0.05. All the data were presented as means ± standard deviations. Graphs were plotted using Origin 2019 Software.

## 4. Conclusions

In summary, LAP exhibited excellent antioxidant activity, including free radical scavenging, scavenging of DPPH radical (IC50 2.91 ± 0.02 mg/mL), hydroxyl radical (IC50 9.95 ± 0.03 mg/mL), and superoxide anion radical (IC50 13.67 ± 0.33 mg/mL). LAP-I and LAP-II had the characteristic properties of peptides and exhibited an irregular amorphous form of crystalline structure. LAP-I and LAP-II exhibited a compact stretch conformation and a folded conformation in the D_2_O solution, respectively. These findings indicated that LAP has excellent antioxidant activity and the potential to be used as a functional food.

## Figures and Tables

**Table 1:** Sequences of active peptides with some antioxidant activity based on LC-MS/MS identification screening.

Components	Amino Acid Sequencing	MW (Da)	Comparison of Scores	Abundance (Strength)	Percentage of Hydrophobic Amino Acids (%)	Charge Number
LAP-I	NHPGQISQ	8.79 × 10^2^	121.29	2.89 × 10^8^	25.00	2
LAP-I	LDAGDGVTH	8.83 × 10^2^	81.32	1.51 × 10^8^	33.33	2
LAP-I	SYELPDGQ	9.1 × 10^2^	60.76	8.80 × 10^7^	25.00	2
LAP-I	MYPGIADR	9.21 × 10^2^	93.56	4.12 × 10^8^	37.50	2
LAP-I	YPGIADRM	9.21 × 10^2^	98.42	7.26 × 10^7^	50.00	2
LAP-I	MEPVLEQS	9.31 × 10^2^	134.81	6.16 × 10^7^	50.00	1
LAP-I	PSADAPMFV	9.33 × 10^2^	89.43	5.01 × 10^7^	77.78	2
LAP-I	GRDLTDYL	9.51 × 10^2^	116.54	1.26 × 10^9^	25.00	2
LAP-I	FIGMESAGIH	1.06 × 10^3^	88.35	1.97 × 10^7^	50.00	2
LAP-I	GRDLTDYLM	1.08 × 10^3^	98.46	1.77 × 10^8^	33.33	2
LAP-I	YLRPHIGESL	1.18 × 10^3^	146.81	8.03 × 10^7^	40.00	2
LAP-I	HVDPDNFRLL	1.22 × 10^3^	102.51	4.72 × 10^8^	50.00	2
LAP-I	GMHGVNEEVFL	1.23 × 10^3^	86.79	2.83 × 10^7^	45.45	2
LAP-I	EHGDSSVPVWSGV	1.35 × 10^3^	129.42	4.86 × 10^8^	38.46	2
LAP-I	LLPVEVPEHIATM	1.45 × 10^3^	82.43	4.68 × 10^7^	69.23	2
LAP-I	KLHVDPDNFRLL	1.47 × 10^3^	105.20	4.02 × 10^8^	50.00	2; 3
LAP-II	MLTL	4.76 × 10^2^	72.01	1.66 × 10^8^	75.00	1
LAP-II	WDDM	5.65 × 10^2^	61.26	1.70 × 10^8^	50.00	1
LAP-II	NDHFVKL	8.71 × 10^2^	109.63	1.15 × 10^9^	42.86	2
LAP-II	HVDPDNFRL	1.11 × 10^3^	102.51	7.65 × 10^7^	44.44	2
LAP-II	MLFPGDFSPE	1.14 × 10^3^	83.75	7.79 × 10^6^	60.00	1
LAP-II	LFPGDFSPEVH	1.24 × 10^3^	77.28	2.71 × 10^7^	54.55	2
LAP-II	GLTPGEHGFHVH	1.29 × 10^3^	83.21	2.78 × 10^7^	33.33	2
LAP-II	LHVDPDNFRLL	1.34 × 10^3^	78.33	8.55 × 10^7^	54.55	2; 3
LAP-II	LRVAPEEHPTLL	1.37 × 10^3^	165.98	9.81 × 10^7^	58.33	2; 3
LAP-I, LAP-II	LTAM	4.34 × 10^2^	63.97	2.67 × 10^7^	75.00	1
LAP-I, LAP-II	VAPEEHPT	8.78 × 10^2^	97.60	1.77 × 10^9^	50.00	2
LAP-I, LAP-II	TPDVHEAW	9.53 × 10^2^	94.54	3.64 × 10^7^	50.00	2
LAP-I, LAP-II	VAPEEHPTL	9.91 × 10^2^	124.89	1.51 × 10^9^	55.56	2
LAP-I, LAP-II	HVDPDNFR	9.98 × 10^2^	138.83	1.08 × 10^7^	37.50	2
LAP-I, LAP-II	APSADAPMFV	1.00 × 10^3^	110.90	6.09 × 10^7^	80.00	2
LAP-I, LAP-II	ITPPLPEQH	1.03 × 10^3^	137.18	3.87 × 10^8^	55.56	2
LAP-I, LAP-II	MYPGIADRM	1.05 × 10^3^	95.91	9.48 × 10^8^	55.56	2
LAP-I, LAP-II	VAPEEHPTLL	1.10 × 10^3^	103.75	6.39 × 10^9^	60.00	2
LAP-I, LAP-II	RVAPEEHPVLL	1.26 × 10^3^	97.64	2.10 × 10^8^	63.64	2; 3

**Note:** Hydrophobic amino acids (HAA) include: Ala (A), Val (V), Leu (L), Ile (I), Phe (F), Trp (W), Met (M), and Pro (P).

**Table 2:** Potential antioxidant active peptides.

Components	Amino Acid Sequencing	Length	MW (Da)	Toxicity	pI
LAP-II	WDDM	4	565.18	Non-toxic	3.57
LAP-I	PSADAPMFV	9	933.43	Non-toxic	3.80

**Table 3:** Chemical shifts of LAP principal component in D_2_O.

LAP-I	Peak	Chemical Displacement	Corresponding Atoms	LAP-II	Peak	Chemical Displacement	Corresponding Atoms
	1	4.35	Ala-H_α_		1	4.16	Val-H_α_
	2	4.17	Val-H_α_		2	4.01	Gly-H_α_
	3	3.92	Gly-H_α_		3	3.91	Ser-H_β_
	4	3.75	Pro-H_δ1_		4	3.77	Pro-H_δ1_
	5	3.63	Pro-H_δ2_		5	3.60	Pro-H_δ2_
	6	3.20	Phe-H_β1_		6	3.22	Phe-H_β_
	7	2.98	Phe-H_β2_		7	3.12	Tyr-H_β_
	8	2.73	Asp-H_β_		8	3.00	Cys-H_β_
	9	2.11	Gln-H_β_		9	2.32	Glu-H_γ_
	10	2.16	Met-H_β_		10	2.09	Gln-H_β_
	11	1.75	Lys-H_β_		11	1.90	Ile-H_β_
	12	1.39	Ala-H_β_		12	1.65	Leu-H_β_
	13	1.18	Ile-H_δ_		13	1.43	Lys-H_γ_
	14	0.93	Val-H_γ_		14	1.34	Ala-H_β_
					15	1.16	Ile-H_γ_
					16	0.95	Val-H_γ_

## Data Availability

No new data were created.

## Sample Availability

Samples of the compounds LAP, LAP-I and LAP-II are available from the authors.
